# Monocyte Chemoattractant Protein-1/CCL2 Produced by Stromal Cells Promotes Lung Metastasis of 4T1 Murine Breast Cancer Cells

**DOI:** 10.1371/journal.pone.0058791

**Published:** 2013-03-18

**Authors:** Teizo Yoshimura, O. M. Zack Howard, Toshihiro Ito, Masaki Kuwabara, Akihiro Matsukawa, Keqiang Chen, Ying Liu, Mingyong Liu, Joost J. Oppenheim, Ji Ming Wang

**Affiliations:** 1 Laboratory of Molecular Immunoregulation, Cancer and Inflammation Program, Center for Cancer Research, National Cancer Institute, Frederick, Maryland, United States of America; 2 Department of Pathology and Experimental Medicine, Graduate School of Medicine, Dentistry and Pharmaceutical Sciences, Okayama University, Okayama, Japan; Kanazawa University, Japan

## Abstract

MCP-1/CCL2 plays an important role in the initiation and progression of cancer. Since tumor cells produce MCP-1, they are considered to be the main source of this chemokine. Here, we examined whether MCP-1 produced by non-tumor cells affects the growth and lung metastasis of 4T1 breast cancer cells by transplanting them into the mammary pad of WT or MCP-1^−/−^ mice. Primary tumors at the injected site grew similarly in both mice; however, lung metastases were markedly reduced in MCP-1^−/−^ mice, with significantly longer mouse survival. High levels of MCP-1 mRNA were detected in tumors growing in WT, but not MCP-1^−/−^ mice. Serum MCP-1 levels were increased in tumor-bearing WT, but not MCP-1^−/−^ mice. Transplantation of MCP-1^−/−^ bone marrow cells into WT mice did not alter the incidence of lung metastasis, whereas transplantation of WT bone marrow cells into MCP-1^−/−^ mice increased lung metastasis. The primary tumors of MCP-1^−/−^ mice consistently developed necrosis earlier than those of WT mice and showed decreased infiltration by macrophages and reduced angiogenesis. Interestingly, 4T1 cells that metastasized to the lung constitutively expressed elevated levels of MCP-1, and intravenous injection of 4T1 cells producing a high level of MCP-1 resulted in increased tumor foci in the lung of WT and MCP-1^−/−^ mice. Thus, stromal cell-derived MCP-1 in the primary tumors promotes lung metastasis of 4T1 cells, but tumor cell-derived MCP-1 can also contribute once tumor cells enter the circulation. A greater understanding of the source and role of this chemokine may lead to novel strategies for cancer treatment.

## Introduction

Leukocytes infiltrate a number of human and mouse cancers [1], [2]. Although the composition of tumor infiltrating leukocytes and the role they play may vary in each tumor, they are generally immunosuppressive and provide a microenvironment that favors tumor growth. Therefore, identifying the mechanisms by which immunosuppressive leukocytes are recruited into tumors is critical and clinically relevant.

Monocyte chemoattractant protein-1 (MCP-1)/CCL2 is a chemokine with potent monocyte chemotactic activity. It was initially purified from the culture supernatant of a human malignant glioma [3] and a monocytic leukemic cell line [4], and was later demonstrated to be identical to the previously described tumor cell-derived chemotactic factor [5]; thus, tumor cells are a source of MCP-1. Earlier animal studies using MCP-1-transfected tumor cells provided both anti- and pro-tumor effects of MCP-1 [6]–[9]; however, accumulating evidence now strongly suggest that the production of MCP-1 by tumors is responsible for the recruitment of immunosuppressive macrophages that promote tumor growth. In a chemically induced skin papilloma model, the number of papillomas in MCP-1-deficient mice was lower compared to that in WT mice [10]. A vital role of MCP-1 in the initiation and progression of colitis-associated colon carcinogenesis was demonstrated by using mice deficient in the MCP-1 receptor CCR2 or MCP-1 blocking agents [11]. In addition, neutralization of MCP-1 resulted in reduced growth of prostate cancer [12]–[14], breast cancer [15] and lung cancer [16] in mice. Thus, MCP-1 is a candidate molecular target of cancer treatment [17].

Tumor tissues contain a variety of non-tumor stromal cells, including fibroblasts, endothelial cells and inflammatory cells. These tumor stromal cells provide the soil in which tumor cells grow, invade and metastasize [18]–[20]. Although tumor cells may be the major source of MCP-1 in the tumor microenvironment as described above, stromal cells also have the capacity to produce MCP-1. In fact, stromal MCP-1 has been implicated in the recruitment of tumor-associated macrophage and subsequent breast cancer progression [21], [22]. However, the relative contribution of stromal cells to the production of MCP-1 and subsequent tumor progression has not been experimentally evaluated.

The 4T1 breast cancer cells were isolated from a spontaneous mammary tumor of a Balb/cC3H mouse. When the cells are orthotopically injected into mammary pads of Balb/c mice, they form tumors and metastasize spontaneously to tissues, such as lung, liver and bone, providing an excellent model to elucidate the mechanisms involved in tumor growth and metastasis [23].

In the present study, we aimed to define the contribution of stromal cell-derived MCP-1 to tumor progression by transplanting 4T1 cells into the mammary pad of WT or MCP-1-deficient (MCP-1^−/−^) mice. Our results indicate that stromal cells are the main source of MCP-1 in 4T1 tumors and stromal cell-derived MCP-1 promotes spontaneous lung metastasis of 4T1 cells. This MCP-1 effect appears to be due to increased recruitment of macrophages and increased angiogenesis in the primary tumor. Interestingly, the expression of MCP-1 was elevated in 4T1 cells that metastasized to the lung and intravenous injection of 4T1 cells producing a high level of MCP-1 resulted in a higher number of tumor foci in the lung of WT and MCP-1^−/−^ mice, suggesting that the tumor cell-derived MCP-1 also promotes lung metastasis by supporting the tumor cell survival, seeding and growth in the lung. A greater understanding of the role for this chemokine in cancer development may lead to novel strategies for cancer treatment.

## Materials and Methods

### Cell lines

4T1 and Lewis lung carcinoma (LLC) cells (ATCC, Manassas, VA) were cultured in RPMI 1640 (Lonza, Walkersville, MD) supplemented by 10% fetal bovine serum (FBS, HyClone, Rogan, UT), 2 mM L-glutamine, penicillin/streptomycin and sodium pyruvate. To obtain cell-free culture supernatants, 1×10^5^ 4T1 or LLC were seeded into 12-well tissue culture plates. After overnight incubation at 37°C, medium was removed and 1 ml of fresh medium was added and incubated at 37°C for 24 hrs in the presence or absence of 1 or 10 ng/ml tumor necrosis factor α (TNFα, R&D Systems, Minneapolis, MN) or 100 ng/ml lipopolysaccharide (LPS, Sigma-Aldrich, St. Louis, MO). Cell-free culture supernatants were obtained and kept at −80°C until use.

### Mice

The generation of MCP-1^−/−^ mice was previously described [24]. The genetic background of MCP-1^−/−^ mice was converted to a Balb/c background using the speed congenics service provided by the Laboratory Animal Sciences Program (LASP), SAIC-Frederick, Frederick, MD. Wild type Balb/c mice were obtained from Charles River, Frederick, MD.

### Tumor transplantation model

4T1 cells were grown to 50 to 80% confluence. Before injection, cells were detached with 0.2% trypsin-EDTA, washed once with medium, three times with PBS and resuspended in PBS at 5×10^5^/ml. Two hundred µl of cell suspension (1×10^5^ cells) were injected into the 2^nd^ mammary pad of female mice. Mice were euthanized at different time points. Tumors were excised and fixed in 10% formalin or frozen in O.C.T. compound. Lungs were perfused with Bouin's solution and then fixed in the same solution. Blood was drawn by heart puncture. Sera were isolated and stored at −80°C until use. For experimental metastasis assays, cells were resuspended in PBS at 2.5×10^6^/ml or 2×10^5^/ml and 0.2 ml (5×10^5^ cells or 4×10^4^ cells) were injected intravenously via tail vein.

To obtain tumor cells from the metastatic tumor nodules in the lung, well isolated tumor nodules were excised, minced, and digested with collagenase VI (Sigma-Aldrich, St. Louis, MO) for 3 hours at room temperature. After removal of tissue debris, cells were rinsed with RPMI 1640 containing 10% FBS, and then plated in a tissue culture plate. Cells were passed for 5 generations at 1∶5 before used. At this stage, the mutated MCP-1 allele was no longer detectable by PCR in tumor cells harvested from the lung of MCP-1^−/−^ mice, indicating that there was no significant contamination by host cells.

### Generation of bone marrow (BM) chimeric mice

Female WT and MCP-1^−/−^ mice were treated with sulfamethoxazole/trimethoprim for two weeks. The mice were irradiated with 800-rads X-rays and then intravenously injected with 5×10^6^ BM cells collected from femurs of either WT or MCP-1^−/−^ mice. Four groups of chiemric mice were generated: WT to WT, MCP-1^−/−^ to WT, WT to MCP-1^−/−^, and MCP-1^−/−^ to MCP-1^−/−^.

To evaluate the efficiency of BM reconstitution, BM cells were collected from femurs of representative mice at the harvest. Genomic DNA was isolated and subjected to Southern blotting.

### Northern and Southern blotting

Northern blot and Southern blot analyses were performed as described [24], [25]. Filters were hybridized at 42°C overnight in 50% formamide, 5× SSC, 5× Denhardt's solution, 50 µg/ml sheared-denatured salmon sperm DNA, 1% SDS, and l×10^6^ dpm/ml of ^32^P-labeled cDNA probe (Perkin Elmer, Cambridge, MA). Filters were washed twice with 2× SSC, 0.5% SDS at room temperature for 15 min and 0.1× SSC, 0.5% SDS at 60°C for 30 min prior to autoradiographic exposure.

### ELISA

The concentrations of MCP-1 were measured in the Lymphokine Testing Laboratory, Clinical Services Program, SAIC-Frederick, Frederick, MD, with an ELISA kit specific for mouse MCP-1 (R&D Systems).

### RT-PCR

Total RNA was isolated by TRI_zol_ (Ambion-Life Technologies, Carlsbad, CA) and treated with TURBO DNase (Ambion-Life Technologies). RT-PCR was performed using the Easy-A One-Tube RT-PCR Kit (Agilent Technologies, Santa Clara, CA) and 100 ng total RNA. Annealing temperature was 52°C for CCR2, and 60°C for β-actin. The primer sequences were as follows: mouse CCR2 forward, 5′-GGTCATGATCCCTATGTGG-3′; mouse CCR2 reverse, 5-CTGGGCACCTGATTTAAAGG-3′ [26], mouse β-actin forward, 5′-TGTGATGGTGGGAATGGGTGAG-3′; mouse β-actin reverse, 5′-TTTGATGTCACGCACGATTTCC-3′.

### Immunohistochemistry

Tissues were embedded in Tissue-Tek OCT compound and then frozen in liquid nitrogen. Seven-micron cryostat sections were prepared, air-dried and then fixed in ice-cold acetone. After treatment with 10% normal goat serum in Tris-buffered saline containing 0.1% Tween 20 (TBS-T) for 20 min at room temperature, tissue sections were incubated with rat monoclonal antibody against F4/80 (AbD Serotec, Raleigh, NC), CD31 (Biolegend, San Diego, CA) or control IgG (5 µg/ml) for 1 h at room temperature. After washing with TBS-T, the sections were incubated with Alexa Flour 647-labelled anti-rat IgG (Invitrogen, Carlsbad, CA) and Alexa Flour 488-labelled anti-pan cytokeratin (eBioscience) for 30 min at room temperature. The expression of each molecule was analyzed by the FV 1000 Confocal microscope system (Olympus, Tokyo, Japan).

### FACS analysis

4T1 cells were grown to 80% confluent in T-75 flasks. Cells were harvested using 0.02% trypsin-EDTA (Life Technologies), washed, and then stained by a monoclonal antibody against CCR2 labeled by PE (R&D Systems). Tumors were excised and digested by collagenase IV for 2 hrs at room temperature. Cell suspension was filtered through a 70 µm nylon mesh, followed by depletion of red blood cells with ACK solution. Cells were stained by monoclonal antibodies against mouse CD45 labeled by PECy5.5 (BD Biosciences, San Jose, CA), CD11b labeled by PE (eBioscience, San Diego, CA), Ly6G labeled by FITC (eBioscience), F4/80 labeled by FITC (eBioscience), or CD206 labeled by APC (BioLegend), and the expression of each molecule was analyzed using a FACScan flow cytometer (BD Biosciences).

### Statistical analysis

Statistical analysis was performed by Student's *t*-test or Log-rank (Mantel-Cox) test, using the GraphPad Prism, Version 4 and 5, GraphPad Software, San Diego, CA. A value of *p<0.05* was considered to be statistically significant.

### Ethics Statement

The experimental protocols of this study were approved by the Frederick National Laboratory for Cancer Research Animal Care and Use Committee, Frederick, MD.

## Results

### Spontaneous lung metastasis of 4T1 cells was reduced in MCP-1^−/−^ mice

**Figure 1:**
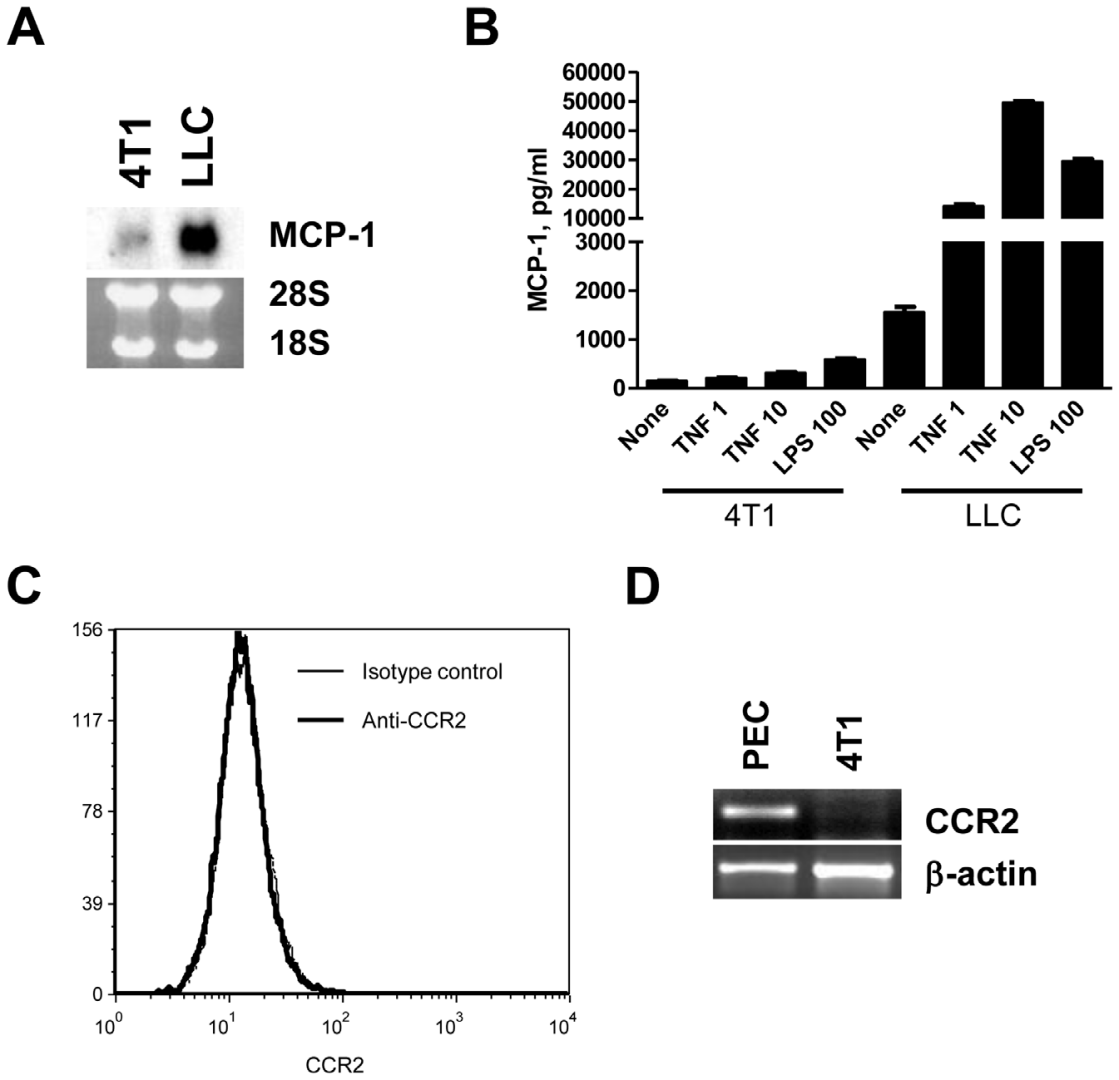
The expression of MCP-1 and CCR2 by 4T1 cells. A. The expression of MCP-1 mRNA by 4T1 cells was examined and compared to that of LLC by Northern blotting. B. The concentration of MCP-1 in the culture supernatants of 4T1 or LLC cells incubated for 24 h with 1 or 10 ng/ml of murine TNFα or 100 ng/ml of LPS or without any stimulus was measured by ELISA. C. The surface expression of CCR2 on 4T1 cells was examined by FACS. D. The expression of CCR2 mRNA in 4T1 cells was examined by RT-PCR. Thioglycollate-induced mouse peritoneal exudates cells were used as control.

We first examined the expression and production of MCP-1 by 4T1 cells. As shown in Figure 1A and B, 4T1 cells constitutively expressed a low level of MCP-1 mRNA and protein. The production of MCP-1 was significantly increased in response to TNF or LPS. However, the level of MCP-1 produced by 4T1 cells was markedly lower than that produced by LLC cells previously shown to produce a high level of MCP-1 [27]. Thus, 4T1 cells have the capacity to produce MCP-1 constitutively or in response to proinflammatory stimuli, but at a low level. The expression of CCR2, the receptor for MCP-1, was not detected in 4T1 cells by either FACS or RT-PCR (Fig. 1C, D). This is consistent with the previous findings that human breast cancer cells also do not express CCR2 [28], [29].

**Figure 2:**
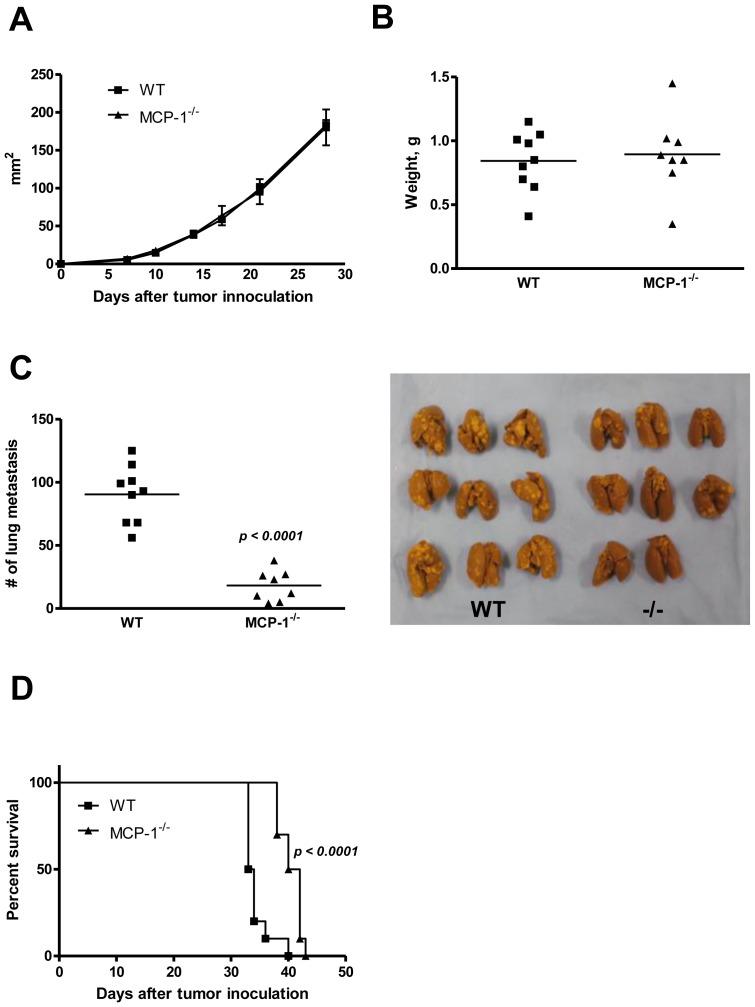
The absence of MCP-1 in tumor stroma reduces the lung metastasis of 4T1 cells and prolongs survival. A. 1×10^5^ 4T1 cells were injected into a mammary pad of WT or MCP-1^−/−^ mice. The size of each primary tumor was measured and the area was calculated. *n = 9* for WT, *n = 8* for MCP-1^−/−^ mice. B. Primary tumors were excised from WT and MCP-1^−/−^ mice 31 days after tumor cell injection and weighed. *n = 9* for WT, *n = 8* for MCP-1^−/−^ mice. C. Mice were euthanized on day 31, and lungs were harvested and fixed in Bouin's solution. The number of metastatic tumor nodules on the surface of lungs of each mouse was counted by eye. *n = 9* for WT, *n = 8* for MCP-1^−/−^ mice. D. Ten thousand 4T1 cells were injected in a mammary pad of each mouse and the survival of each mouse was examined. *n = 8* for WT, *n = 8* for MCP-1^−/−^ mice.

After intra-mammary injection of 4T1 cells, tumor size increased at a similar rate in both WT and MCP-1^−/−^ mice at the injected sites and there was no difference in their weight at 4 weeks (Fig. 2A, B). Interestingly, however, the number of metastatic tumor nodules in the lung of MCP-1^−/−^ mice was significant lower compared to that of WT mice (Fig. 2C). MCP-1^−/−^ mice also survived longer than WT mice (Fig. 2D). These results indicated that MCP-1 regulates not only carcinogenesis, as previously reported [11], but also metastatic spread. Our results also strongly suggested that the main source of MCP-1 in 4T1 tumors was non-tumor cells in tumor stroma.

### Non-tumor cells are the major source of MCP-1 in 4T1 tumor

**Figure 3:**
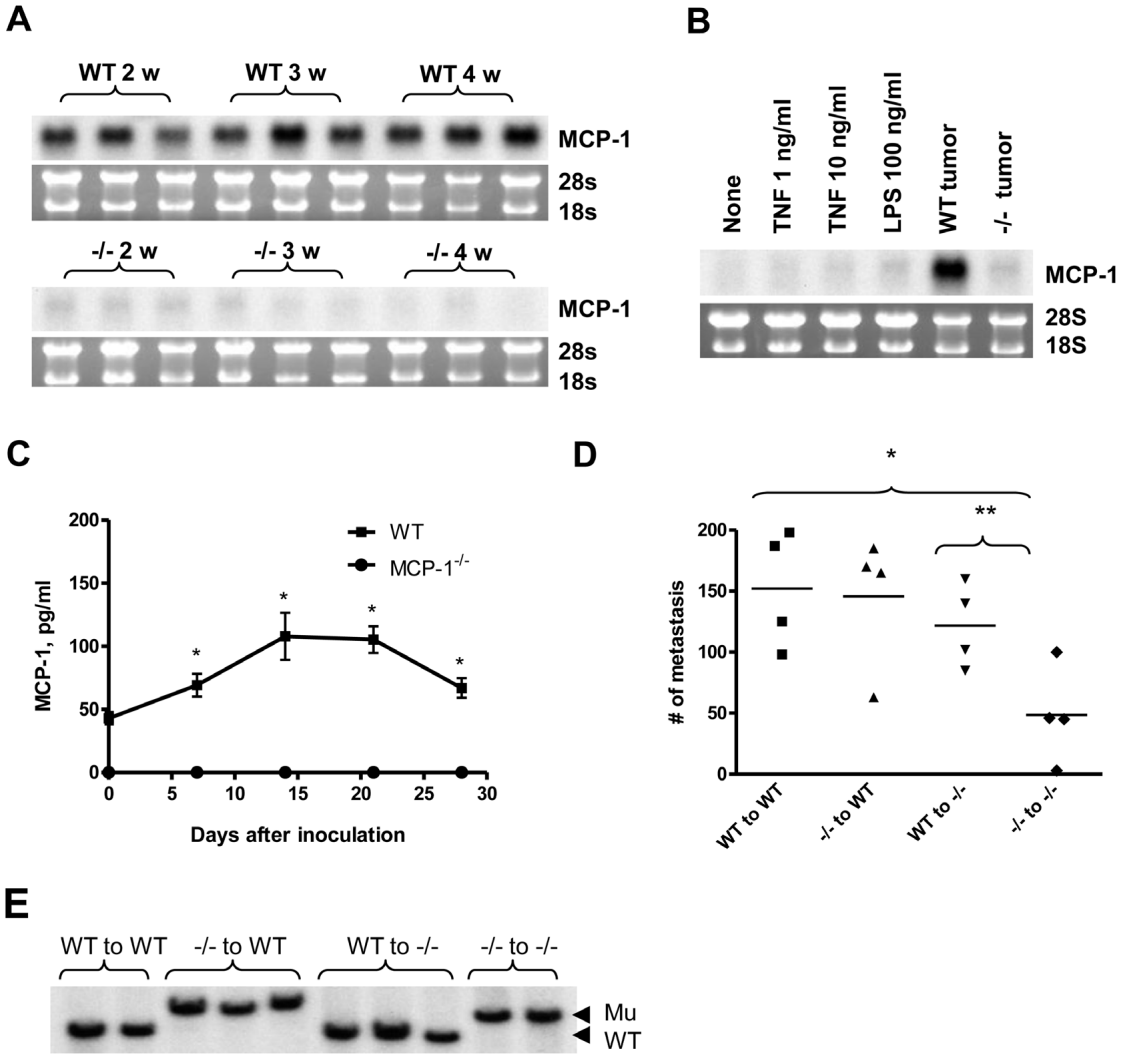
Non-tumor cells in tumor stroma are the major source of MCP-1. A. 1×10^5^ 4T1 cells were injected into a mammary pad of WT or MCP-1^−/−^ mice. The expression of MCP-1 mRNA in tumors of WT or MCP-1^−/−^ mice was examined by Northern blotting. All RNA samples were run on a single agarose gel, blotted to a single membrane. The membrane was hybridized to ^32^P-labeled cDNA probe and exposed to a X-ray film. B. The level of MCP-1 mRNA expression in a WT and MCP-1^−/−^ tumor was compared with that of 4T1 cells incubated in vitro with or without proinflammatory stimuli by Northern blotting. C. The serum MCP-1 concentration of tumor-bearing mice 7, 14, 21 or 28 days after tumor cell inoculation was measured by ELISA. *n = 3* per each group at each time point. *p<0.05. D. 1×10^5^ 4T1 cells were injected into a mammary pad of each mouse. All mice were euthanized 4 weeks after the injection and lungs were inflated with Bouin's solution and fixed in the same solution. The number of metastatic tumor nodules on the surface of the lung of each mouse was counted by eye. *n = 4* for each group. E. BM cells were collected from femurs of mice belonging to each group and genomic DNA was extracted. Ten µg of genomic DNA was digested with *Bam*HI and then subjected to Southern blot analysis. The 7.5-kb WT allele and the 5.5-kb mutant allele are indicated by arrow heads.

We next determined the cellular source of MCP-1 in 4T1 tumors. As shown in Figure 3A, MCP-1 mRNA was readily detectable in tumors of WT mice at 2, 3 and 4 weeks after tumor cell injection, but it was hardly detectable in tumors of MCP-1^−/−^ mice (Fig. 3A). The level of MCP-1 mRNA detected in tumors of WT mice was clearly higher than that of 4T1 cells stimulated in vitro with TNF or LPS (Fig. 3B). The level of MCP-1 mRNA expressed in tumors of MCP-1^−/−^ mice was comparable to that of in vitro stimulated 4T1 cells (Fig. 3B).

The level of MCP-1 in sera of tumor-bearing mice was also examined. Sera from non-tumor-bearing WT mice contained approximately 43 pg/ml of MCP-1. After injection of 4T1 cells, serum MCP-1 level significantly increased at 1 week, peaked at 3 week and then decreased at 4 week in WT mice. In contrast, MCP-1 was not detectable in the sera of tumor-bearing MCP-1^−/−^ mice (Fig. 3C). Taken together, these results supported our assumption that the main source of MCP-1 was not tumor cells, but non-tumor stromal cells.

### MCP-1 produced by both hematopoietic and non-hematopoietic cells promotes lung metastasis of 4T1 cells

To identify the cell type(s) contributing to the production of MCP-1 and subsequent lung metastasis of 4T1 cells, we generated bone marrow chimera mice and examined the lung metastasis of 4T1 cells. Consistent with our results shown above, the numbers of metastatic tumors in the lung of MCP-1^−/−^ mice that received MCP-1^−/−^ BM cells (−/− to −/−) were significantly lower than those in the lung of WT mice that received WT BM cells (WT to WT) (Fig. 3D). The numbers of metastatic tumors detected in the lung of WT mice that received MCP-1^−/−^ BM cells (−/− to WT) and MCP-1^−/−^ mice that received WT BM cells (WT to −/−) were similar to those in WT mice that received WT BM cells (WT to WT). Interestingly, the numbers of metastatic tumors detected in the lung of MCP-1^−/−^ mice that received WT BM cells (WT to −/−) were markedly higher than those in the lung of MCP-1^−/−^ mice that received MCP-1^−/−^ BM cells (−/− to −/−) (Fig. 3D).

To exclude the possibility that the lack of reduction in lung metastasis detected in WT mice that received MCP-1^−/−^ BM cells (−/− to WT) was due to the incomplete BM chimerism, we collected BM cells from the representative mice used in this experiment, isolated genomic DNA and performed Southern blotting. As shown in Figure 3E, BM cells from all mice showed the genotype of transplanted BM cells, indicating that BM chimerism was complete. Thus, MCP-1 produced by both hematopoietic and non-hematopoietic cells plays a critical role in promoting the lung metastasis of 4T1 cells, and MCP-1 produced by BM-derived cells, likely macrophages, is sufficient.

### Tumor formation of intravenously injected 4T1 cells was not reduced in the lung of MCP-1^−/−^ mice

It has been reported that early changes in the local microenvironment at the metastatic site, termed the “premetastatic niche”, dictate the pattern of metastatic spread [30]. The production of chemokines at the metastatic site can also regulate metastasis via interaction with corresponding chemokine receptors expressed on the surface of tumor cells [29], [31]. In our model, MCP-1 is absent in non-tumor cells at both primary and metastatic sites, and it was unclear whether the absence of MCP-1 in the lung affected the capacity of 4T1 cells to metastasize to the lung. To examine this possibility, we injected 1×10^5^ or 4×10^4^ 4T1 cells intravenously into WT or MCP-1^−/−^ mice and compared the numbers of tumor nodules in the lungs after 2 weeks. Similar levels of tumor formation were detected in the lung of both WT and MCP-1^−/−^ mice (data not shown), supporting the conclusion that MCP-1 produced in the primary tumors, but not in the lung, was responsible for the lung metastasis of 4T1 cells.

### MCP-1-deficiency in tumor stroma resulted in reduced macrophage infiltration, deficient angiogenesis, and early tumor necrosis

**Figure 4:**
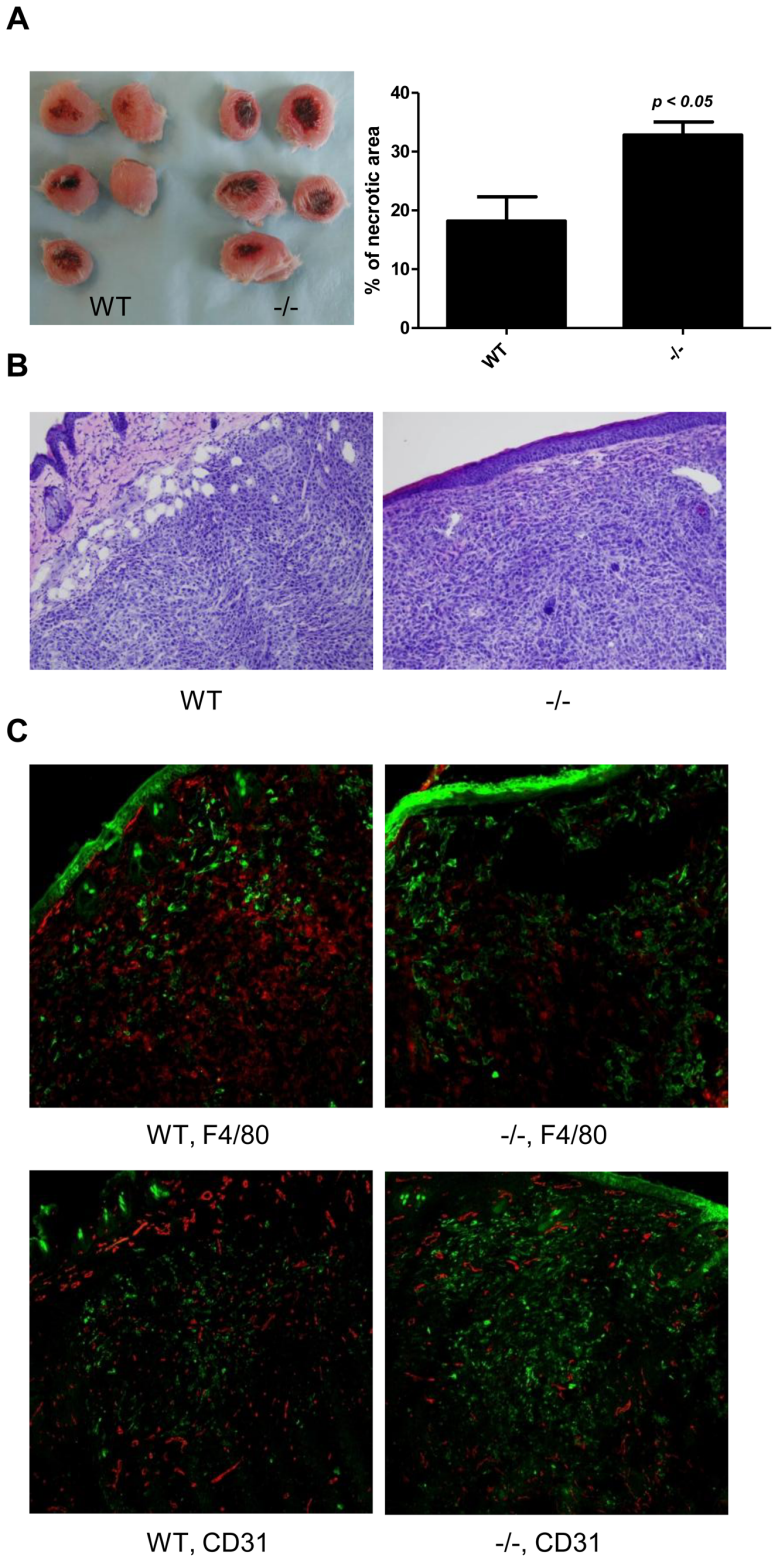
MCP-1-deficiency in tumor stroma results in early necrosis, reduced macrophage infiltration and reduced angiogenesis. A. 1×10^5^ 4T1 cells were injected into a mammary pad of WT or MCP-1^−/−^ mice. Primary tumors were excised two weeks after 4T1 cell injection and fixed in formalin. Tissue sections were prepared from the center of each tumor, stained by H&E, and the area of necrosis was measured. % of necrotic area was calculated by using a formula, area of necrosis/area of tumor×100. *n = 5* for each group. B. H&E section of tumor tissue away from necrosis. C. Immunohistochemical examination of tumor sections. Green, cytokeratin; Red, F4/80 or CD31.

As demonstrated above, our results strongly suggested that MCP-1 produced in the primary tumor microenvironment, but not in the lung, affected the spontaneous lung metastasis of 4T1 cells. To define the role of MCP-1 in the lung metastasis, we examined the macroscopic and histological features of primary tumors growing in WT and MCP-1^−/−^ mice. Although the sizes of the primary tumors were similar in both WT and MCP-1^−/−^ mice as shown in Figure 2A, we consistently observed early necrosis of tumors of MCP-1^−/−^ mice and the percentage of the necrotic area of tumors of MCP-1^−/−^ mice was significantly higher than that of WT mice (Fig. 4A). The formation of necrotic lesion was detected as early as 1 week after tumor cell injection into MCP-1^−/−^ mice at which time necrotic lesion was not observed in the tumors of WT mice.

Histologically, more intense infiltration of leukocytes was observed in the subcutaneous area of the tumors in MCP-1^−/−^ mice (Fig. 4B). Immunohistochemical examination revealed that the infiltration of F4/80 cells was markedly reduced in tumors of MCP-1^−/−^ mice (Fig. 4C). The reduction in the percentage of F4/80 positive cells in the tumors of MCP-1^−/−^ mice was also found by flow cytometry analysis (Table 1). In a separate experiment, we examined the cell population that expresses CD206, a marker for M2 macrophages. There was no significant difference in the percentage of CD206-positive cells among F4/80-positive cells (68.7±3.2% in WT, *n = 3*, vs 62.1±3.9% in MCP-1^−/−^ mice, *n = 3*). Conversely, the percentage of CD45^+^CD11b^+^Ly6G^low^ cells (MDSCs) increased in the tumors of MCP-1^−/−^ mice (Table 1). A similar shift to neutrophil-rich leukocyte infiltration was previously found when different tumor cell lines were injected into CCR2^−/−^ mice [32], [33]. Finally, positive staining with anti-CD31 Ab was also decreased in tumors of MCP-1^−/−^ mice. Taken together, our results indicated that MCP-1 deficiency in tumor stromal cells resulted in the reduced macrophage infiltration and subsequent reduced angiogenesis, likely leading to the reduced exit of tumor cells into blood vessels necessary for lung metastasis.

**Table 1:** Analysis of tumor-infiltrating leukocyte subsets 3 weeks after 4T1 cell inoculation.

Cell types	Markers	WT (%)	MCP-1^−/−^ (%)
Myeloid cells	CD45^+^CD11b^+^	90.6±0.5	94.3±0.5**
MDSCs	CD45^+^CD11b^+^Ly6G^low^	21.5±1.2	26.0±0.5*
Macrophages	CD45^+^CD11b^+^F4/80^+^	15.8±2.0	6.1±0.8***

Tumors were excised and digested by collagenase IV for 2 hrs at room temperature. Cell suspensions were filtered and red blood cells were depleted with ACK solution. Cells were stained by monoclonal antibodies against mouse CD45 labeled by PECy5.5, CD11b labeled by PE, Ly6G labeled by FITC or F4/80 labeled by FITC, and the expression of each molecule was analyzed using a FACScan flow cytometer. *n = 3*,

* *p<0.05*,

** *p<0.01*,

*** *p<0.001*.

### Elevated MCP-1 production by 4T1 cells has no effect on their lung metastasis but supports their seeding and growth in the lung

**Figure 5:**
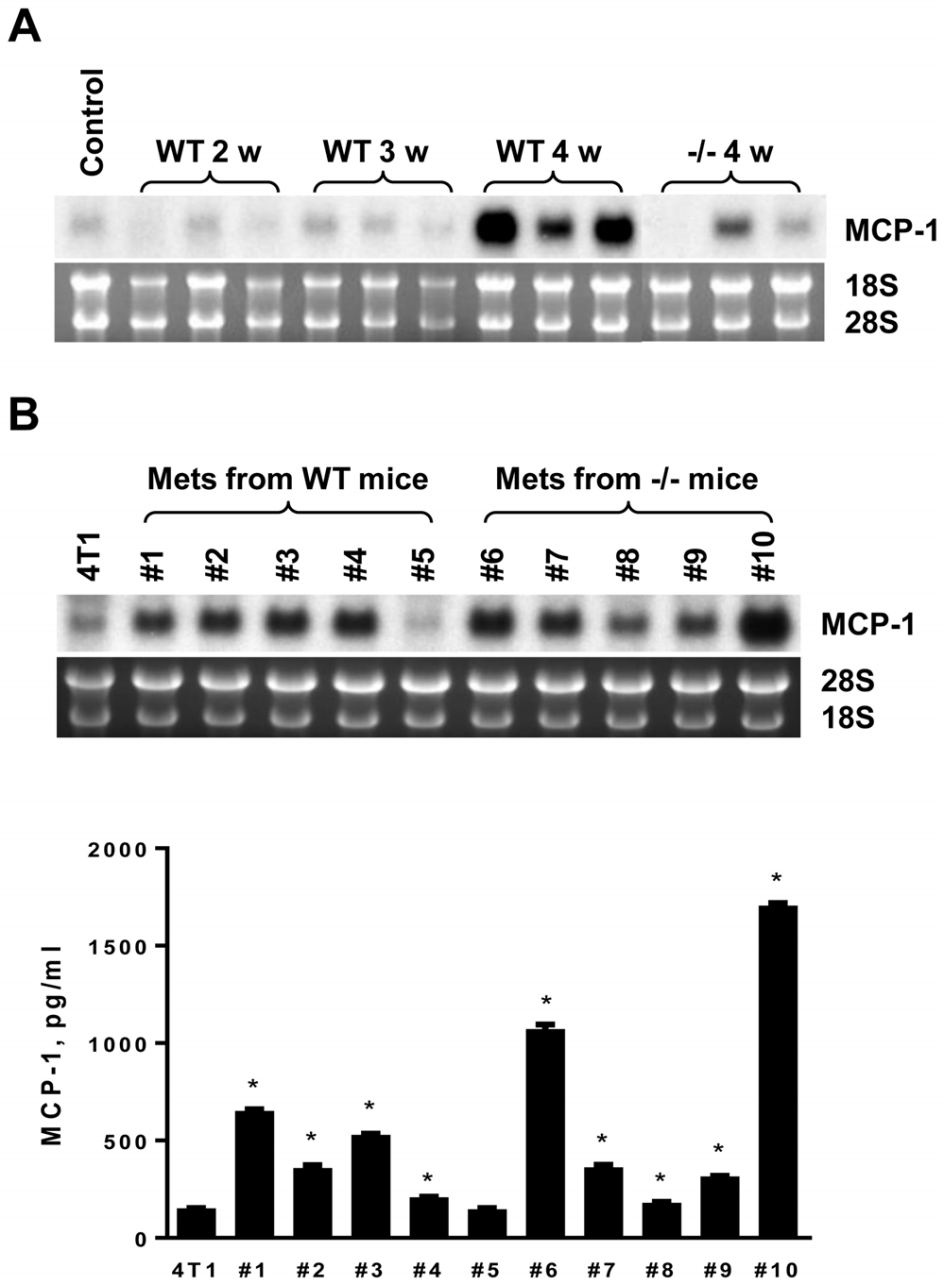
4T1 cells that metastasized to the lung express higher level of MCP-1. A. 1×10^5^ 4T1 cells were injected into a mammary pad of WT or MCP-1^−/−^ mice. Total RNA was extracted from the lung of tumor-bearing WT or MCP-1^−/−^ mice and the expression of MCP-1 mRNA was examined by Northern blotting. B. Total RNA was extracted from 4T1 cells obtained from metastatic lung tumors of WT or MCP-1^−/−^ mice and the expression of MCP-1 mRNA was evaluated by Northern blotting (upper panel). 1×10^5^ cells were seeded into 12-well plates and incubated at 37°C overnight. Medium was replaced by 1 ml fresh medium and incubated for an additional 24 hrs. Cell-free culture supernatants were collected and the concentrations of MCP-1 were measured by ELISA (lower panel). * *p<0.01, n = 2*.

To examine the cellular source of MCP-1 at the metastatic site, we evaluated the expression of MCP-1 in the lung of tumor-bearing WT and MCP-1^−/−^ mice. As shown in Figure 5A, a low level of MCP-1 mRNA was detectable in the lung of non-tumor-bearing WT mouse. The level of MCP-1 mRNA was not altered in the lung of tumor-bearing WT mice up to 3 weeks after injection, but a significant increase was detected at 4 weeks when metastatic tumor nodules were readily visible. As expected, in the lung of tumor-bearing MCP-1^−/−^ mice, MCP-1 mRNA was undetectable up to 3 weeks; however, unlike the primary tumors, the expression of MCP-1 mRNA was clearly detectable at 4 weeks, indicating that the expression of MCP-1 was upregulated in tumor cells.

To determine whether up-regulated MCP-1 expression in tumor cells that metastasized to the lung was constitutive or in response to stimuli, we isolated tumor cells from well-isolated metastatic tumor nodules in the lung of WT or MCP-1^−/−^ mice, depleting non-tumor cells and then examined the expression of MCP-1 mRNA in the resulting tumor cells. As shown in Figure 5B, the majority of tumor cell clones recovered from the metastatic lung tumors of both WT and MCP-1^−/−^ mice constitutively expressed higher levels of MCP-1 mRNA and produced higher levels of MCP-1than the original 4T1 cells, leading to the hypothesis that higher MCP-1 expression by tumor cells may also support lung metastasis.

**Figure 6:**
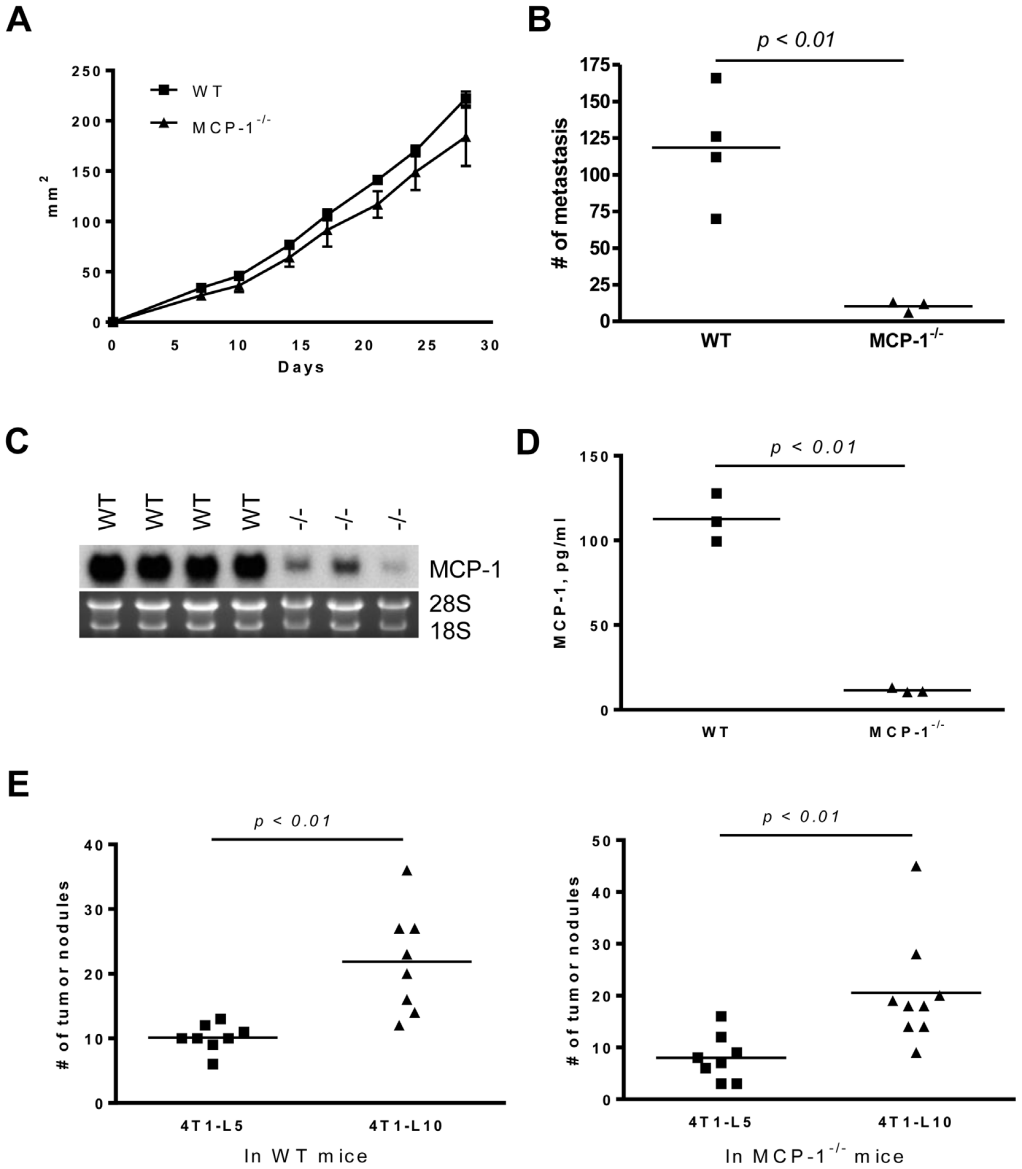
Increased MCP-1 expression in 4T1 cells has no effect on spontaneous lung metastasis in MCP-1^−/−^ mice, but increases lung metastasis after intravenous injection. A. 1×10^5^ 4T1-L10 cells were injected into a mammary pad of WT or MCP-1^−/−^ mice. The size of each primary tumor was measured and the area was calculated. *n = 4* for WT, *n = 3* for MCP-1^−/−^ mice. B. 1×10^5^ 4T1-L10 cells were injected into a mammary pad of WT or MCP-1^−/−^ mice. All mice were euthanized 4 weeks after the injection and the number of metastatic tumor nodules on the surface of each lung was counted by eye. C. Total RNA was extracted from each tumor and the expression of MCP-1 mRNA was examined by Northern analysis. Ten µg of total RNA was used. D. Sera were collected 2 weeks after the injection of 4T1-L10 cells and MCP-1 concentrations were measured by ELISA. E. 4T1-L5 or 4T1-L10 cells (5×10^4^ cells in 0.2 ml PBS) were intravenously injected into WT (left panel) or MCP-1^−/−^ mice (right panel). Two weeks later, mice were euthanized and the number of metastatic tumor nodules on the lung was counted. The results are the summary of two independent experiments. *n = 8* for WT, and *n = 8* (4T1-L5) or *n = 9* (4Y1-L10) for MCP-1^−/−^ mice.

We tested the hypothesis using a subclone of 4T1 cells (4T1-L10, clone #10 in Fig. 5B) constitutively producing a 10-fold higher level of MCP-1 compared to the original 4T1 cells or another subclone 4T1-L5 producing a low level of MCP-1 (clone #5 in Fig. 5B). We first injected 4T1-L10 cells into the mammary pad of WT and MCP-1^−/−^ mice. 4T1-L10 cells grew at the same rate as the original 4T1 cells in both mice at the injected sites (Fig. 6A). All tumors growing in MCP-1^−/−^ mice developed marked necrosis 2 weeks after the injection, whereas only one of 4 tumors growing in WT mice showed a small necrotic lesion. This was similar to the finding when the original 4T1 cells were injected in those mice (Fig. 4A). Contrary to our hypothesis, the number of metastatic tumor nodules in the lung of MCP-1^−/−^ mice remained low (Fig. 6B). Although MCP-1 mRNA could be detected readily in the primary 4T1-L10 tumors growing in MCP-1^−/−^ mice, the levels of MCP-1 mRNA in the primary tumors were still much lower than those detected in the 4T1-L10 tumors growing in WT mice (Fig. 6C). The serum MCP-1 concentrations were also low in tumor-bearing MCP-1^−/−^ mice 2 weeks after the injection (Fig. 6D). These results indicated that non-tumor cells were the main source of MCP-1 also in 4T1-L10 tumors and that tumor cell-derived MCP-1 had little effect on the spontaneous lung metastasis of 4T1 cells, likely due to the insufficient angiogenesis.

Next we intravenously injected 4T1-L5 or 4T1-L10 cells into WT or MCP-1^−/−^ mice. As shown in Figure 6E, intravenous injection of 4T1-L10 cells resulted in an approximately 2-fold higher number of tumor nodules in the lung of WT (left panel) and MCP-1^−/−^ mice (right panel), suggesting that tumor cell-derived MCP-1 may contribute to the lung metastasis by supporting their survival and seeding in the lung once tumor cells invade blood vessels.

## Discussion

Metastasis is a multistep process, involving tumor cell migration, intravasation and survival in circulation, extravasation and colonization in distant tissues [34]. In order for tumor cells to initiate the metastatic process, tumor cells must acquire the ability to migrate and invade, likely through a sequence of genetic and epigenetic alterations. In addition, non-tumor cells must provide a microenvironment that favors the seeding, survival and growth of tumor cells. In IL-1 receptor-deficient [35] or CSF-1-deficient mice [36] in which tumor microenvironment was markedly altered by reduced infiltration of leukocytes, spontaneous lung metastasis of breast cancer cells was significantly reduced. Thus, cooperation between tumor cells and non-tumor cells in tumor stroma is critical for tumor metastasis. In the present manuscript, we demonstrated that MCP-1 produced by stromal cells promotes lung metastasis of 4T1 breast cancer cells by providing a microenvironment that favors metastasis.

The production of MCP-1 was previously examined in human breast cancer tissues. Both tumor cells and stromal cells in the tumor, such as lymphoreticular cells, fibroblasts, endothelial cells and macrophages, were positive for MCP-1 by immunohistochemistry [21], [22], [37]. The expression of MCP-1 in tumor cells showed a significant correlation with the level of thymidine phosphorylation and membrane type 1-matrix metalloproteinase expression [21], whereas MCP-1 expression in macrophages in tumor stroma correlated with the level of microvessel density and vessel invasion of tumor cells. These observations of human breast cancer tissues are consistent with our results obtained with the 4T1 mouse breast cancer model, indicating that our model is clinically valid.

Studies of bone marrow chimeras indicated that both hematopoietic and non-hematopoietic cells contributed to the MCP-1 production, and that MCP-1 produced by either cell population was sufficient to promote lung metastasis of 4T1 cells. Among hematopoietic cells, macrophages have been shown to facilitate the malignant transformation of mammary tumors and their pulmonary metastasis. For example, in the absence of CSF-1, the transformation and growth of the primary tumors in polyoma middle T antigen (PyMT) mice was not affected, but the recruitment of macrophages and the progression to malignancy and the metastatic spread were significantly reduced [36]. In vivo chemotaxis assay suggested that epidermal growth factors and CSF-1, but not MCP-1, were the chemotactic factors responsible for the recruitment of macrophages into tumors [38], [39]. As noted above, MCP-1 was originally purified from tumor cell lines as a chemoattractant responsible for the recruitment of TAMs [3]–[5]. A direct correlation between the expression of MCP-1 and macrophage infiltration was previously detected in human breast cancer tissues and neutralization of MCP-1 significantly decreased the number of tumor-infiltrating macrophages [21], [22], [37]. In our study, the infiltration of macrophages into tumors was markedly reduced in the absence of MCP-1, indicating that MCP-1 is one of the critical chemoattractants for the recruitment of macrophages into tumors, such as breast cancer, and that macrophages themselves are one of the major sources of MCP-1.

Our studies revealed that the primary tumors of MCP-1^−/−^ mice develop necrosis significantly earlier than those of WT mice and that the blood vessel formation was markedly reduced in tumors of MCP-1^−/−^ mice. MCP-1 can indirectly promote angiogenesis by recruiting monocytes, which in turn release angiogenic factors, such as VEGF [37], [40]. MCP-1 can also directly promote angiogenesis by activating the migration and Ets-1 transcription factor in endothelial cells [41], [42], and by recruiting smooth muscle cells toward endothelial cells [43]. Thus, it is likely that MCP-1-deficiency in tumor stroma resulted in reduced angiogenesis and intravasation of tumor cells, leading to reduced lung metastasis.

The expression of MCP-1 by stromal cells suggests that they are activated by the product(s) of 4T1 cells. 4T1 cells were previously shown to express transcripts for CSF-1, 2 and 3, and CSF-1 has the capacity to induce MCP-1 production in leukocytes, such as macrophages [44]. When 4T1 cells were inoculated into the mammary pad of interleukin -1 receptor (IL-1R)-deficient mice, the levels of cytokines, including IL-6, TNFα and MCP-1, in the tumor tissue were significantly reduced and both tumor progression and lung metastasis were reduced [35]. In another study, co-culture of mouse macrophages with the human MDA-MB-231 breast cancer cells in vitro upregulated MCP-1 production by mouse macrophages [22]. These previous findings suggest that breast cancer cells release mediators, such as CSF-1 and IL-1, to activate tumor-infiltrating macrophages to produce MCP-1. We recently observed that the culture supernatant of 4T1 cells was capable of inducing MCP-1 production in mouse macrophages, independent of IL-1, IL-6, TNFα, or CSF-1 (Yoshimura, unpublished data). Identifying the molecule(s) responsible for the induction of MCP-1 in macrophages by cancer cells may provide a new means to target tumor microenvironment.

It was previously demonstrated that at metastatic sites, a distinct population of metastasis-associated macrophages promotes the extravasation, seeding and persistent growth of tumor cells [45], [46]. Qian et al. recently identified the phenotype of these macrophages as Gr1^+^ inflammatory macrophages expressing CCR2. Neutralization of MCP-1 derived from either tumor cells or stromal cells blocked the recruitment of inflammatory monocytes, inhibited lung metastasis of breast cancer cells and prolonged the survival of tumor-bearing mice [46]. In our study, the lung metastasis of intravenously injected 4T1 cells in MCP-1^−/−^ mice was comparable to that in WT mice; thus circulating 4T1 cells appear to metastasize to the lung independently of MCP-1 produced by lung tissues. However, tumor cells are heterogeneous and tumor cells producing higher levels of MCP-1 may have an advantage in metastasizing to the lung. In fact, the levels of MCP-1 expressed in 4T1 cells recovered from metastatic tumor nodules in the lung of WT or MCP-1^−/−^ mice were higher than that of the original in vitro cultured 4T1 cells. Furthermore, intravenous injection of 4T1-L10 cells producing a 10-fold higher level of MCP-1 resulted in increased lung metastasis in both WT and MCP-1^−/−^ mice. Thus, tumor cells with the capacity to produce higher levels of MCP-1 show better survival and seeding in a remote metastatic site. Additional studies are necessary to determine the mechanisms by which MCP-1 expression is constitutively upregulated in metastasized 4T1 cells.

We previously reported that the expression and production of MCP-3/CCL7, which is highly similar to MCP-1 and attracts monocytes through CCR2 [47], [48], were up-regulated in our MCP-1^−/−^ mice [24]; therefore, the low level lung metastases detected in MCP-1^−/−^ mice might be due to the increased MCP-3 production. As expected, the expression of MCP-3 mRNA was markedly higher in tumors of MCP-1^−/−^ mice than those of WT mice. The serum MCP-3 levels were also significantly elevated in MCP-1^−/−^ mice and the sums of MCP-1 and MCP-3 serum concentrations in tumor-bearing WT and MCP-1^−/−^ mice were comparable (data not shown). These results indicated that increased MCP-3 production in MCP-1^−/−^ mice was not sufficient to compensate for the loss of MCP-1 and strongly suggested that MCP-3 does not play a significant role in the lung metastasis of 4T1 cells. We did not examine the production of MCP-5/CCL12, another CCR2 ligand. Interestingly, the number of metastatic tumor nodules in the lung of CCR2^−/−^ mice was not significantly different from that of MCP-1^−/−^ mice (data not shown). MCP-5 was recently shown not to be involved in the recruitment of metastatic macrophages in a mouse breast cancer model [46]. Therefore, MCP-5 does not appear to play a role in the lung metastasis of 4T1 cells. Thus, although all three MCP's bind CCR2 and induce monocyte migration in vitro, MCP-1 is the responsible CCR2 ligand in vivo, in particular, in tumor metastasis.

In contrast to its prometastatic role, MCP-1 was recently reported to also promote an anti-metastatic host response [49]. Gr1^+^ neutrophils accumulated in the lung prior to the arrival of metastatic cells in mouse models of breast cancer, including mouse 4T1 model. Interestingly, neutrophils were entrained to inhibit metastatic seeding in the lung by generating H_2_O_2_ and tumor-secreted MCP-1 was a critical mediator of anti-metastatic entrainment of G-CSF-stimulated neutrophils. In our study, the metastasis of 4T1 cells to the lung was markedly reduced in MCP-1^−/−^ mice, indicating that MCP-1-mediated neutrophil entraining does not play a significant role. Nevertheless, we found that an increased number of neutrophils infiltrated in the marginal areas of primary tumors of MCP-1^−/−^ mice, which may represent anti-tumor progression and anti-metastasis host responses in the absence of MCP-1.

In conclusion, we have demonstrated that non-tumor stromal cells play a critical role in the spontaneous lung metastasis of 4T1 breast cancer cells by providing a high level of MCP-1 which facilitates angiogenesis by recruiting pro-angiogenic macrophages and perhaps by directly acting on endothelial cells. Tumor cell-derived MCP-1 has no effect on spontaneous lung metastasis of 4T1 cells; however, it can contribute to the lung metastasis by supporting tumor cell survival and seeding in the lung once tumor cell invade blood vessels. Thus, MCP-1 produced by stromal cells and tumor cells orchestrates the metastatic process of 4T1 cells. Targeting MCP-1 may reduce lung metastasis of breast cancer cells.

## References

[1] Mantovani A, Allavena P, Sica A, Balkwill F (2008) Cancer-related inflammation. Nature 454: 436–444. 18650914 10.1038/nature07205

[2] Grivennikov SI, Greten FR, Karin M (2010) Immunity, inflammation, and cancer. Cell 140: 883–899. 20303878 10.1016/j.cell.2010.01.025 PMC2866629

[3] Yoshimura T, Robinson EA, Tanaka S, Appella E, Kuratsu J, et al (1989) Purification and amino acid analysis of two human glioma cell-derived monocyte chemoattractants. J Exp Med 169: 1449–1459. 2926329 10.1084/jem.169.4.1449 PMC2189237

[4] Matsushima K, Larsen CG, DuBois GC, Oppenheim JJ (1989) Purification and characterization of a novel monocyte chemotactic and activating factor produced by a human myelomonocytic cell line. J Exp Med 169: 1485–1490. 2926331 10.1084/jem.169.4.1485 PMC2189236

[5] Bottazzi B, Colotta F, Sica A, Nobili N, Mantovani A (1990) A chemoattractant expressed in human sarcoma cells (tumor-derived chemotactic factor, TDCF) is identical to monocyte chemoattractant protein-1/monocyte chemotactic and activating factor (MCP-1/MCAF). Int J Cancer 45: 795–797. 2182547 10.1002/ijc.2910450436

[6] Bottazzi B, Walter S, Govoni D, Colotta F, Mantovani A (1992) Monocyte chemotactic cytokine gene transfer modulates macrophage infiltration, growth, and susceptibility to IL-2 therapy of a murine melanoma. J Immunol 148: 1280–1285. 1737940

[7] Hirose K, Hakozaki M, Nyunoya Y, Kobayashi Y, Matsushita K, et al (1995) Chemokine gene transfection into tumour cells reduced tumorigenicity in nude mice in association with neutrophilic infiltration. Br J Cancer 72: 708–714. 7669585 10.1038/bjc.1995.398 PMC2033873

[8] Rollins BJ, Sunday ME (1991) Suppression of tumor formation in vivo by expression of the JE gene in malignant cells. Mol Cell Biol 11: 3125–3131. 2038321 10.1128/mcb.11.6.3125-3131.1991 PMC360158

[9] Yamashiro S, Takeya M, Nishi T, Kuratsu J, Yoshimura T, et al (1994) Tumor-derived monocyte chemoattractant protein-1 induces intratumoral infiltration of monocyte-derived macrophage subpopulation in transplanted rat tumors. Am J Pathol 145: 913–921. 7943176 PMC1887319

[10] Moore RJ, Owens DM, Stamp G, Arnott C, Burke F, et al (1999) Mice deficient in tumor necrosis factor-α are resistant to skin carcinogenesis. Nat Med 5: 828–831. 10395330 10.1038/10552

[11] Popivanova BK, Kostadinova FI, Furuichi K, Shamekh MM, Kondo T, et al (2009) Blockade of a chemokine, CCL2, reduces chronic colitis-associated carcinogenesis in mice. Cancer Res 69: 7884–7892. 19773434 10.1158/0008-5472.CAN-09-1451

[12] Loberg RD, Ying C, Craig M, Day LL, Sargent E, et al (2007) Targeting CCL2 with systemic delivery of neutralizing antibodies induces prostate cancer tumor regression in vivo. Cancer Res 67: 9417–9424. 17909051 10.1158/0008-5472.CAN-07-1286

[13] Lu Y, Cai Z, Xiao G, Keller ET, Mizokami A, et al (2007) Monocyte chemotactic protein-1 mediates prostate cancer-induced bone resorption. Cancer Res 67: 3646–3653. 17440076 10.1158/0008-5472.CAN-06-1210

[14] Li X, Loberg R, Liao J, Ying C, Snyder LA, et al (2009) A destructive cascade mediated by CCL2 facilitates prostate cancer growth in bone. Cancer Res 69: 1685–1692. 19176388 10.1158/0008-5472.CAN-08-2164 PMC2698812

[15] Lu X, Kang Y (2009) Chemokine (C-C motif) ligand 2 engages CCR2+ stromal cells of monocytic origin to promote breast cancer metastasis to lung and bone. J Biol Chem 284: 29087–29096. 19720836 10.1074/jbc.M109.035899 PMC2781454

[16] Fridlender ZG, Kapoor V, Buchlis G, Cheng G, Sun J, et al (2011) CCL2 blockade inhibits lung cancer tumor growth by altering macrophage phenotype and activating CD8+ cells. Am, J Respir Cell Mol Biol 44: 230–237. 20395632 10.1165/rcmb.2010-0080OC PMC3049234

[17] Garber K (2010) First results for agents targeting cancer-related inflammation. J Natl Cancer Inst 101: 1110–1112. 10.1093/jnci/djp266 19671776

[18] Sund M, Kalluri R (2009) Tumor stroma derived biomarkers in cancer. Cancer Metastasis Rev 28: 177–183. 19259624 10.1007/s10555-008-9175-2 PMC4476244

[19] Albini A, Sporn MB (2007) The tumour microenvironment as a target for chemoprevention. Nature Rev 7: 139–147. 10.1038/nrc2067 17218951

[20] Weiseman BS, Werb Z (2002) Stromal effects on mammary gland development and breast cancer. Science 296: 1046–1049. 12004111 10.1126/science.1067431 PMC2788989

[21] Saji H, Koike M, Yamori T, Saji S, Seiki M, et al (2001) Significant correlation of monocyte chemoattractant protein-1 expression with neovascularization and progression of breast carcinoma. Cancer 92: 1085–1091. 11571719 10.1002/1097-0142(20010901)92:5<1085::aid-cncr1424>3.0.co;2-k

[22] Fujimoto H, Sangai T, Ishii G, Ikehara A, Nagashima T, et al (2009) Stromal MCP-1 in mammary tumors induces tumo-associated macrophage infiltration and contributes to tumor progression. Int J Cancer 125: 1276–1284. 19479998 10.1002/ijc.24378

[23] Heppner GH, Miller FR, Shekhar PVM (2000) Nontransgenic models of breast cancer. Breast Cancer Res 2: 331–334. 11250725 10.1186/bcr77 PMC138654

[24] Takahashi M, Galligan C, Tessarollo L, Yoshimura T (2009) Monocyte chemoattractant protein-1 (MCP-1), not MCP-3, is the primary chemokine required for monocyte recruitment in mouse peritonitis induced with thioglycollate or zymosan A. J Immunol 183: 3463–3471. 19641140 10.4049/jimmunol.0802812 PMC7371094

[25] Yoshimura T, Johnson DG (1993) cDNA cloning and expression of guinea pig neutrophil attractant protein-1 (NAP-1): NAP-1 is highly conserved in guinea pig. J Immunol 151: 6225–6236. 7504015

[26] Boring L, Gosling J, Chensue SW, Kunkel SL, Farese RV Jr, et al (1997) Impaired monocyte migration and reduced type 1 (Th1) cytokine responses in C-C chemokine receptor 2 knockout mice. J Clin Invest 100: 2552–2561. 9366570 10.1172/JCI119798 PMC508456

[27] Stathopoulos GT, Psallidas I, Moustaki A, Moschos C, Kollintza A, et al (2008) A central role for tumor-derived monocyte chemoattractant protein-1 in malignant pleural effusion. J Natl Cancer Inst 100: 1464–1476. 18840818 10.1093/jnci/djn325

[28] Davidson B, Dong HP, Holth A, Berner A, Risberg B (2008) The chemokine receptor CXCR4 is more frequently expressed in breast compared to other metastatic adenocarcinomas in effusions. Breast J 14: 476–482. 18657145 10.1111/j.1524-4741.2008.00625.x

[29] Müller A, Homey B, Soto H, Ge N, Catron D, et al (2001) Involvement of chemokine receptors in breast cancer metastasis. Nature 410: 50–56. 11242036 10.1038/35065016

[30] Kaplan RN, Rafii S, Lyden D (2006) Preparing the “soil”: the premetastatic niche. Cancer Res 66: 11089–11093. 17145848 10.1158/0008-5472.CAN-06-2407 PMC2952469

[31] Wang JM, Chertov O, Proost P, Li JJ, Menton P, et al (1998) Purification and identification of chemokines potentially involved in kidney-specific metastasis by a murine lymphoma variant: induction of migration and NFkappaB activation. Int J Cancer 75: 900–907. 9506536 10.1002/(sici)1097-0215(19980316)75:6<900::aid-ijc13>3.0.co;2-6

[32] Sawanobori Y, Ueha S, Kurachi M, Shimaoka T, Talmadge JE, et al (2008) Chemokine-mediated rapid turnover of myeloid-derived suppressor cells in tumor-bearing mice. Blood 111: 5457–5466. 18375791 10.1182/blood-2008-01-136895

[33] Pahler JC, Tazzyman S, Erez N, Chen YY, Murdoch C, et al (2008) Plasticity in tumor-promoting inflammation: impairment of macrophage recruitment evokes a compensatory neutrophil response. Neoplasia 10: 329–340. 18392134 10.1593/neo.07871 PMC2288539

[34] Chaffer CL, Weinberg RA (2011) A perspective on cancer cell metastasis. Science 331: 1559–1564. 21436443 10.1126/science.1203543

[35] Bunt SK, Yang L, Sinha P, Clements VK, Leips J, et al (2007) Reduced inflammation in the tumor microenvironment delays the accumulation of myeloid-derived suppressor cells and limits tumor progression. Cancer Res 67: 10019–10026. 17942936 10.1158/0008-5472.CAN-07-2354 PMC4402704

[36] Lin EY, Nguyen AV, Russell RG, Pollard JW (2001) Colony-stimulating factor promotes progression of mammary tumors to malignancy. J Exp Med 193: 727–739. 11257139 10.1084/jem.193.6.727 PMC2193412

[37] Ueno T, Toi M, Saji H, Muta M, Bando H, et al (2000) Significance of macrophage chemoattractant protein-1 in macrophage recruitment, angiogenesis, and survival in human breast cancer. Clin Cancer Res 6: 3282–3289. 10955814

[38] Wyckoff J, Wang W, Lin EY, Wang Y, Pixley F, et al (2004) A paracrine loop between tumor cells and macrophages is required for tumor cell migration in mammary tumors. Cancer Res 64: 7022–7029. 15466195 10.1158/0008-5472.CAN-04-1449

[39] Pollard JW (2008) Macrophages define the invasive microenvironment in breast cancer. J Leukoc Biol 84: 623–630. 18467655 10.1189/jlb.1107762 PMC2516896

[40] Sunderkötter C, Steinbrink K, Goebeler M, Bhardwaj R, Sorg C (1994) Macrophages and angiogenesis. J Leukoc Biol 55: 410–422. 7509844 10.1002/jlb.55.3.410

[41] Salcedo R, Ponce ML, Young HA, Wasserman K, Ward JM, et al (2000) Human endothelial cells express CCR2 and respond to MCP-1: direct role of MCP-1 in angiogenesis and tumor progression. Blood 96: 34–40. 10891427

[42] Stamatovic SM, Keep RF, Mostarica-Stojkovic M, Andjelkovic AV (2006) CCL2 regulates angiogenesis via activation of Ets-1 transcription factor. J Immunol 177: 2651–2661. 16888027 10.4049/jimmunol.177.4.2651

[43] Arderiu G, Peña E, Aledo R, Juan-Babot O, Badimon L (2011) Tissue factor regulates microvessel formation and stabilization by induction of chemokine (C-C motif) ligand 2 expression. Arterioscler Thromb Vasc Biol 31: 2607–2615. 21868706 10.1161/ATVBAHA.111.233536

[44] Irvine KM, Andrews MR, Fernandez-Rojo MA, Schroder K, Burns CJ, et al (2009) Colony-stimulating factor-1 (CSF-1) delivers a proatherogenic signal to human macrophages. J Leukoc Biol 85: 278–288. 19004987 10.1189/jlb.0808497

[45] Qian B, Deng Y, Im JH, Muschel RJ, Zou Y, et al (2009) A distinct macrophage population mediates metastatic breast cancer cell extravasation, establishment and growth. PLoS One 4: e6562. 19668347 10.1371/journal.pone.0006562 PMC2721818

[46] Qian BZ, Li J, Zhang H, Kitamura T, Zhang J, et al (2011) CCL2 recruits inflammatory monocytes to facilitate breast-tumour metastasis. Nature 475: 222–225. 21654748 10.1038/nature10138 PMC3208506

[47] Franci C, Wong LM, Van Damme J, Proost P, Charo IF (1995) Monocyte chemoattractant protein-3, but not monocyte chemoattractant protein-2, is a functional ligand of the human monocyte chemoattractant receptor. J Immunol 154: 6511–6517. 7759884

[48] Jia T, Serbina NV, Brandl K, Zhong MX, Leiner IM, et al (2008) Additive roles for MCP-1 and MCP-3 in CCR2-mediated recruitment of inflammatory monocytes during *Listeria monocytogenes* infection. J Immunol 180: 6846–6853. 18453605 10.4049/jimmunol.180.10.6846 PMC2386263

[49] Granot Z, Henke E, Comen EA, King TA, Norton L, et al (2011) Tumor entrained neutrophils inhibit seeding in the premetastatic lung. Cancer Cell 20: 300–314. 21907922 10.1016/j.ccr.2011.08.012 PMC3172582

